# Beyond interviews and focus groups: a framework for integrating innovative qualitative methods into randomised controlled trials of complex public health interventions

**DOI:** 10.1186/s13063-019-3439-8

**Published:** 2019-06-06

**Authors:** Katy Davis, Nicole Minckas, Virginia Bond, Cari Jo Clark, Tim Colbourn, Sarah J. Drabble, Therese Hesketh, Zelee Hill, Joanna Morrison, Oliver Mweemba, David Osrin, Audrey Prost, Janet Seeley, Maryam Shahmanesh, Esther J. Spindler, Erin Stern, Katrina M. Turner, Jenevieve Mannell

**Affiliations:** 1 Institute for Global Health, University College London, 30 Guilford Street, London, WC1N 1EH UK; 2 Department of Global Health and Development, Faculty of Public Health and Policy, London School of Hygiene and Tropical Medicine, 15-17 Tavistock Place, London, WC1H 9SH UK; 3 Zambart House, School of Public Health, University of Zambia, Box 50697, Lusaka, 10101 Zambia; 4 Rollins School of Public Health, Emory University, 1518 Clifton Road NE, Claudia Nance Rollins Building, 7033, Atlanta, GA 30322 USA; 5 School of Health and Related Research, University of Sheffield, 30 Regent St, Sheffield, S1 4DA UK; 6 Department of Health Promotion and Education, School of Public Health, Ridgeway Campus University of Zambia, Box 50110, Lusaka, 10101 Zambia; 7 Heilbrunn Department of Population and Family Health, Columbia University Mailman School of Public Health, 722 W 168th St, New York, 10032 NY USA; 8 Population Health Sciences, University of Bristol, 39 Whatley Road, Bristol, BS8 2PS UK; 9 The National Institute for Health Research Collaboration for Leadership in Applied Health Research and Care West (NIHR CLAHRC West), University Hospitals Bristol NHS Foundation Trust, Bristol, UK

**Keywords:** Qualitative method, Complex intervention, Public health, RCTs, Innovation

## Abstract

**Background:**

Randomised controlled trials (RCTs) are widely used for establishing evidence of the effectiveness of interventions, yet public health interventions are often complex, posing specific challenges for RCTs. Although there is increasing recognition that qualitative methods can and should be integrated into RCTs, few frameworks and practical guidance highlight which qualitative methods should be integrated and for what purposes. As a result, qualitative methods are often poorly or haphazardly integrated into existing trials, and researchers rely heavily on interviews and focus group discussions. To improve current practice, we propose a framework for innovative qualitative research methods that can help address the challenges of RCTs for complex public health interventions.

**Methods:**

We used a stepped approach to develop a practical framework for researchers. This consisted of (1) a systematic review of the innovative qualitative methods mentioned in the health literature, (2) in-depth interviews with 23 academics from different methodological backgrounds working on RCTs of public health interventions in 11 different countries, and (3) a framework development and group consensus-building process.

**Results:**

The findings are presented in accordance with the CONSORT (Consolidated Standards of Reporting Trials) Statement categories for ease of use. We identify the main challenges of RCTs for public health interventions alongside each of the CONSORT categories, and potential innovative qualitative methods that overcome each challenge are listed as part of a Framework for the Integration of Innovative Qualitative Methods into RCTs of Complex Health Interventions. Innovative qualitative methods described in the interviews include rapid ethnographic appraisals, document analysis, diary methods, interactive voice responses and short message service, community mapping, spiral walks, pair interviews and visual participatory analysis.

**Conclusions:**

The findings of this study point to the usefulness of observational and participatory methods for trials of complex public health interventions, offering a novel contribution to the broader literature about the need for mixed methods approaches. Integrating a diverse toolkit of qualitative methods can enable appropriate adjustments to the intervention or process (or both) of data collection during RCTs, which in turn can create more sustainable and effective interventions. However, such integration will require a cultural shift towards the adoption of method-neutral research approaches, transdisciplinary collaborations, and publishing regimes.

## Background

In this article, we argue that many of the challenges facing randomised controlled trials (RCTs) of complex public health interventions can be addressed through the integration of a diverse toolbox of qualitative methods and we propose a framework of potential methods. RCTs are widely used for establishing evidence of the effectiveness of interventions, yet public health interventions are often complex, posing specific challenges for RCTs which qualitative research can help address. Qualitative research methods offer important insights for the evaluation of health interventions and increasingly are being used as part of RCTs [1–3]. Research investigating the potential value of qualitative methods has highlighted their role in facilitating the transferability of interventions, improving external validity, providing a more nuanced understanding of contexts and processes, and improving delivery of interventions. All of these ultimately increase the utility of evidence generated during RCTs [2–5].

Pope and Mays [6] claim that qualitative research is a “prerequisite of good quantitative research, particularly in areas that have received little previous investigation” (page 42). It can provide insight into the contextual circumstances of the implementation, delivery and evaluation of interventions [7]. Qualitative research is particularly valuable for evaluating complex health interventions, which are often found in public health. A complex intervention is composed of different interacting components that are multifaceted and socially mediated [8, 9]. The complexity resides in the variety of behaviours required by participants in the intervention, the groups or organisational levels targeted, the potentially large number of outcomes, and the degree of flexibility permitted by the intervention [10], all of which are associated with emergent phenomena that are difficult to predict. All of these factors mean that complex health interventions are often challenging to define and therefore reproduce [11, 12]. Moreover, complex interventions are increasingly recognised as belonging to “open” systems in ways that make planned interventions and their surrounding context difficult to disentangle using conventional RCT designs [13]. Qualitative methods can help address these challenges by “reaching the parts that other methods cannot reach” ([6], page 42), including understanding non-linear causality, complex relationships between context and interventions, and dynamic or emergent results [6, 13].

Although there is increasing recognition that qualitative methods can and should be integrated into RCTs, few frameworks and practical guidance highlight which qualitative methods should be integrated and for what purposes. As a result, qualitative methods often are poorly or haphazardly integrated into existing trials, contributing to variation in the quality of qualitative research used alongside trials [1, 5]. This is evidenced by the lack of explicit reference to how qualitative findings have been used to interpret or help explain quantitative trial results in published articles [3, 14] and is compounded by what O’Cathain et al. [3] refer to as the “add-on status of qualitative research” (page 121) in quantitative health research.

### Challenges of using RCTs to evaluate complex health interventions

Although RCTs are currently perceived as the “gold standard” in global health evaluation, their limitations are well documented [15, 16]. Power et al. [11] point to several implementation trials that have lost internal validity when failing to consider the broader contexts in which they were carried out [17–19]. Other scholars acknowledge a disconnect between the recommendation that interventions be standardised to ensure valid measurement of trial outcomes and the very concept of complex healthcare systems [14].

These critiques of RCTs and standardised approaches to intervention are well recognised [10]. Recent Medical Research Council (MRC) guidelines on developing and evaluating complex interventions suggest a range of designs to address the challenges of conventional experimental trials, including cluster randomised and stepped wedge designs; preference trials, including “*Wennberg*” and “*Rucker*” designs, which base randomisation on individuals’ preferences; and randomised consent “*Zelen*” designs, which randomly assign individuals prior to taking consent [20]. Others have discussed the need for “adaptive” trial designs whereby changes are made to a randomisation protocol or the trial outcomes on the basis of information collected while the trial is under way [21, 22]; however, little mention has been made of the potential for qualitative research or patient/community preferences to influence such adaptations. Moving away from conventional approaches to clinical RCTs, “pragmatic trials” attempt to establish the effectiveness of interventions under routine conditions instead of rigid experimental design [23]. Hawe et al. [24] argue that, in pragmatic trials, the evaluation process and function can be standardised while the form of the intervention is made adaptable to context. Pragmatic trials are designed to reflect the real-world heterogeneity of participants and situations, often keeping exclusion criteria to a minimum and not necessarily requiring blinding [25]. A pragmatic RCT approach is seen as providing an opportunity to evaluate not only the intervention but also its interaction with the complex social, cultural, legal and ethical context, which may be as important as the intervention itself [25].

However, as large-scale experimental studies, both adaptive and pragmatic RCTs remain resource-intensive, posing challenges to produce reliable and accessible evidence of generalisable impact and relevance to the targeted population [26–28]. The persistent use of trials to evaluate complex interventions in community settings has led in some cases to rather expensive evaluations that have failed to produce any significant findings [24]. Discussing the research process as whole, Chalmers and Glasziou argue that there is 85% cumulative waste in the production and reporting of research evidence as a result of correctable problems [26]. An important aspect of this waste relates to inappropriate methods, which contribute to incomplete reporting of trial outcomes and the need to measure new outcomes not part of the original plan [28].

Attempting to address these limitations and drawing on the advantages of qualitative methods discussed previously, we propose a framework for integrating qualitative methods into quantitative evaluations of complex health interventions. We aim to answer the question: *how can innovative qualitative methods address the challenges of RCTs as an evaluation methodology?* In this article, we draw on key informant interviews with 23 researchers involved in RCTs to identify how qualitative methods can be used to address the challenges that the evaluation of complex health interventions raises for RCT methodologies.

### Using qualitative methods to address the challenges

Methodological frameworks for the use of qualitative methods alongside quantitative intervention evaluation methods are currently limited in justifying the need for qualitative methods. Mixed methods evidence about the use of qualitative methods alongside trials centres on time-related or “temporal” frameworks. Along these lines, Sandelowski [29] highlights how qualitative research can be incorporated as formative evaluations before the trial begins, process evaluations during the trial, or impact evaluations after an intervention [29]. Creswell et al. build on Sandelowski’s work and argue that it is the purpose of the qualitative data collection that defines whether the “before”, “during” or “after” model should be used. In the stages before a trial begins, qualitative research is said to be most useful for defining research topics while ensuring the intervention’s relevance and appropriateness to the populations of interest [30]. Nested within a trial, qualitative process evaluations may indicate the reasons behind an intervention failure, unexpected consequences, or success [8]. Finally, after a trial is complete, the use of qualitative methods is said to help “interpret quantitative findings and question underlying theory and assumptions to better inform future hypotheses and intervention design” [31] (page 714). Frameworks within this group include the MRC Framework [8], which also divides the process of developing and evaluating an intervention in a trial into time-related phases.

There are, however, alternatives to temporal frameworks. Flemming et al. [30] have developed a framework that focuses on the contribution of qualitative research to specific RCT processes, including planning, recruitment, randomisation, conduct, attrition and implementation. In line with the work of Flemming et al., O’Cathain et al. [3] argue that temporal frameworks contribute to a lack of clarity about when the qualitative research actually takes place as part of an RCT. Synthesising the use of qualitative research within specific trials across 296 studies, they produce an alternative framework that includes five broad categories: the intervention being tested, the trial design and conduct, outcomes, measures used within the trial, and the intervention studied.

However, neither temporal nor process frameworks go much beyond a description of qualitative research and its characteristics. Despite methodological discussions about when to collect qualitative data and for what purpose, there has been little discussion to date of what qualitative methods might be most useful for complementing quantitative RCT data in the evaluation of complex health interventions. As such, existing frameworks have not contributed to broadening the discussion around the inherent value of having a diverse selection of qualitative methods to draw on for RCTs, and how much greater innovation and diversity of methods could be drawn on to strengthen what are often unrealised attempts at mixed methods. Thus, there is a need for trialists to “look beyond focus groups and interviews” ([3], page 50) and expand the extremely limited diversity of qualitative methods currently being used [3, 32].

In this article, we offer a new framework developed from expert researchers’ accounts of using qualitative methods as a component of the trial. Rather than offering an alternative to temporal or process frameworks, our aim is to highlight the diversity of qualitative methods that can be used to address many of the major challenges complex health interventions pose for RCTs. We suggest innovative examples of qualitative methods to demonstrate the adaptive and extensive nature of qualitative research as part and parcel of RCTs.

## Methods

We followed a stepped approach to develop a practical framework for researchers selecting qualitative methods to use within RCTs of public health interventions. Our aim was to cover a range of potential trial designs, including individually randomised, cluster randomised and stepped wedge. Our approach consisted of (1) a review of the qualitative methods mentioned in the health literature, (2) in-depth interviews with academics from different methodological backgrounds (quantitative and qualitative) working on RCTs of public health interventions, and (3) framework development and group consensus-building.

The inclusion of qualitative and quantitative researchers in the study provided an opportunity to gather methodological perspectives on the inherent value of using qualitative methods within trials. The use of in-depth interviews to gather academics’ opinions and experiences provided a means of overcoming some of the power dynamics involved in qualitative and quantitative research paradigms and minimising the influence of group dynamics [33]. The participants in the study were also invited to be co-authors on the final paper to ensure that the framework was developed and refined through a group decision-making process.

### Step 1: Review of qualitative methods used in RCTs

An initial list of innovative qualitative methods was derived through searching the published literature using Scopus. We used the term “innovative” to refer to qualitative methods other than standard interviews and focus group discussions. The “sources” section of Scopus was searched on the 8th of November 2017 for journals that contained “qualitative” (*n* = 23) and “method” (*n* = 160) in their titles. Journals were assessed for relevance on the basis of their focus on qualitative methodologies and English language. This search produced a list of 25 journals (Table 1). Two further journals that had been identified by Wiles et al. [28] were added.

**Table 1:** Final list of journals searched for innovative qualitative methods

Behaviour Research Methods	
BMS Bulletin of Sociological Methodology/Bulletin de Methodologie Sociologique	
Cultural Studies - Critical Methodologies	
Field Methods	
Forum Qualitative Sozialforschung	
International Journal of Qualitative Studies in Education	
International Journal of Qualitative Studies on Health and Well-being	
International Journal of Research and Method in Education	
International Journal of Social Research Methodology: Theory and Practice	
Journal of Mixed Methods Research	
Methodology	
Organizational Research Methods	
Psychological Methods	
Qualitative Health Research	
Qualitative Inquiry	
Qualitative Report	
Qualitative Research	
Qualitative Research in Psychology	
Qualitative Research Journal	
Qualitative Social Work	
Qualitative Sociology Review	
Qualitative Research in Sport, Exercise and Health	
Sociological Methodology	
Sociological Methods and Research	
The International Journal of Qualitative Methods	
Quality and Quantity	
Methodological Innovations Online	

The website catalogues of these journals were then searched with the terms “innov*, new, novel, emerg*” as previously used in the methodology by Wiles et al. [34]. These search terms were used in order to identify methods considered innovative or new to qualitative methodologists. This produced 654 search results which were exported to Endnote (Thomson Reuters). Journal articles from before 2008 were removed to capture methodological discussions over the past 10 years. Abstracts were screened for mention of qualitative methodologies beyond interviews and focus group discussions or for discussions of methodological innovations. This produced 127 full text articles of interest, which were further screened for mention or discussion of methods. A list of methods was subsequently compiled (Table 2).

**Table 2:** List of qualitative methods identified in the literature

Art	Art workshops, collage, dance, decoupage, drama, drawing, drawing method of storytelling, graffiti, imprography, imitation games, improvisation, magazine collage, mural, music, painting, performative methodologies, role-play, scenario workshop, sculpture, sketching, street theatre.
Mapping	Argument maps, body-mapping, circle map, concept mapping, digital mind maps, digital traces, emotion map, process maps, egocentric sociograms, social network analysis, spider diagrams
Multimedia	Avatar representation, bio-photographic elicitation interviews, video recordings, computer mediated communication, conversation audio recordings, documentary film, head-mounted cameras, spatial montage, skype interviews, twitter data, video shadowing, videoconference focus groups, videovoice.
Narrative	Audio diaries, scrapbook diaries, biographic narrative, biographic workshop, creative non-fiction, creative writing, digital storytelling, dramatic writing, experimental writing, fiction writing, memory box, narrative poetry, poetic reflection, scroll-back method, stimulated recall.
Visual	Visual dialogues, flash card activity, interpretation panels, mood boards, photo elicitation, photographic portraits as autobiography, photography exhibition, photovoice, social vignettes.

### Step 2: Key informant identification and in-depth interviews

#### Identifying key informants

**Fig. 1:**
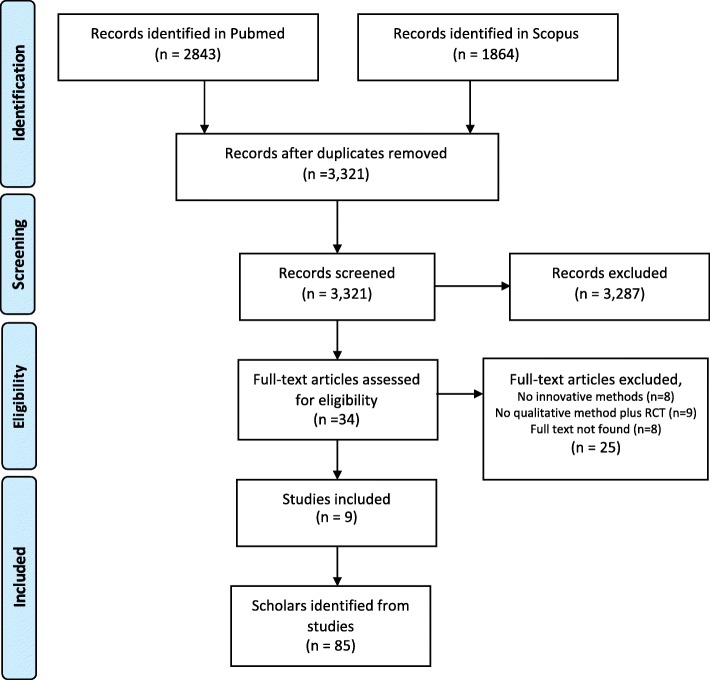
Flow diagram of studies to identify scholars

The list of methods generated during step 1 was adapted to produce a search strategy to identify scholars working in mixed methods. We searched Scopus and Medline for articles that mentioned any of the qualitative methods from step 1 alongside RCTs. Emphasis was placed on methods that were not interviews or focus group discussions without a theoretical rationale mentioned in their design. This produced a final selection of nine journal articles (Fig. 1). Authors of these articles were reviewed to categorise them as potential key informants according to whether they were primarily publishing qualitative or quantitative research, their discipline, geographic location, and research position [35].

The initial list of scholars identified in the literature (*n* = 85) was reduced to first and last authors only (*n* = 18) and supplemented by professional research networks (*n* = 25) and snowballing (*n* = 15). The final list of experts (*n* = 58) and their categorisation was used to ensure maximum heterogeneity in the group composition as a means of encouraging and heightening creativity in the decision-making process that followed [36].

We invited 58 experts to participate in the study. In the invitation sent by email, they were given an information sheet and consent form and asked to confirm their consent to participate with an explicit email response. Interview participants were offered the opportunity to contribute as authors of this paper under the condition that they provide input into each draft. A final tally of 23 experts agreed to participate in the in-depth interviews (Table 3).

**Table 3:** Demographic characteristics of respondents

	Number	Percentage
Total	23	100
Primary methodological approach		
Quantitative	4	17
Qualitative	10	43
Both	9	39
Research position		
Researcher (including associate and senior)	6	26
Lecturer (including senior)	11	48
Professor	6	26
Primary area of expertise		
Gender and HIV	2	9
Maternal and child health	4	17
Health services research	4	17
Behavioural sciences	1	4
Anthropology	5	22
Epidemiology	4	17
Sexual and reproductive health	2	9
Methodology	1	4
Region of affiliation		
Europe	17	74
North America	4	17
Africa	2	9
Primary region of research		
Europe	3	13
North America	1	4
Asia	6	26
Africa	8	35
Not specific	4	17
Method of identification		
Literature review	3	13
Network search	13	57
Snowballing	7	30

Participants had experience working in China, Ethiopia, India, Malawi, Nepal, Rwanda, South Africa, Uganda, the UK, the US, and Zambia. Anonymity was maintained between participants throughout the study up until the paper-writing stage, and participant co-authors were not involved in the analysis of the raw data. The study received ethical approval from the University College London (UCL) research ethics committee (number 12449/001).

#### Data collection through in-depth interviews

A topic guide was used for the interview with a broad set of questions about the inherent value of using qualitative methods within RCTs, methods being used to address intervention or evaluation challenges, and some of the challenges faced when mixing methods. Interviews were conducted by phone or Skype and lasted 50 min on average. The interviews were audio-recorded and transcribed. Transcripts were entered into NVivo (QSR International, Melbourne, Australia) for qualitative data analysis.

### Step 3: Framework development and group consensus process

We conducted a thematic analysis of the interview data by using a framework approach [37]. We adapted a version of the Consolidated Standards of Reporting Trials (CONSORT) Statement [38] as our framework in an attempt to move beyond current discussions of how qualitative research can add value to trials and highlight the indispensable nature of qualitative methods as a complement to quantitative research methodologies. CONSORT is recognised as an essential tool for reporting trial outcomes and widely used by medical journals and international editorial groups [39]. This provided an ideal starting point for appealing to quantitative researchers using a well-recognised framework. To adapt the CONSORT Statement for our purposes, our research team (KD, NM and JM) conducted a rapid review of the literature on the main methodological challenges of using RCTs to evaluate complex public health interventions and organised these challenges according to the CONSORT Statement’s main headings (Table 4) [40–45]. Because of our focus on developing a framework for qualitative methods rather than reporting trial outcomes, not all of the CONSORT Statement categories were relevant. We then merged relevant CONSORT headings into final categories according to their key challenges.

**Table 4:** Challenges identified in the literature by CONSORT Statement’s main headings [40–45]

Section/Topic	Challenge	Threat	Adapted CONSORT categories
Introduction			
Background and objectives	Ensure relevance of the problem to the context	Can reduce engagement	Background and setting
Methods			
Participants	Guarantee representativeness of the sample	Selection bias: can reduce internal and external validity	Recruitment and enrolment
Intervention	Ensure cultural acceptability and practical feasibility	Adherence and withdrawal bias: can reduce internal validity	Intervention design and compliance
	Improve fidelity to the intervention	Adherence bias: can reduce internal validity	
Outcome	Enhance reliability and quality of data	Information (instrument, recall and social desirability) bias: can reduce internal validity	Data collection
	Maintain objectivity during the data collection process		
Sample size	Develop efficient recruitment methods	Selection bias: can reduce internal and external validity	Recruitment and enrolment
Randomisation	Guarantee comparability between groups	Selection and confounding bias: can reduce internal and external validity	Randomisation and allocation
Allocation	Reduce manipulation during the allocation	Selection and confounding bias: can reduce internal and external validity	
Implementation	Improve fidelity to the intervention	Adherence bias: can reduce internal validity	Intervention design and compliance
Blinding	N/A	N/A	–
Statistical methods	N/A	N/A	–
Result			
Participant flow	Minimise the number of participants leaving the study	Attrition and confounding bias: can reduce internal validity	Participant follow-up
Recruitment	Develop efficient recruitment methods	Selection bias: can reduce internal and external validity	Recruitment and enrolment
Baseline data and number analysed	N/A	N/A	–
Outcomes and ancillary analyses	Identify the mechanisms underpinning the effect of the intervention	N/A	Analysis and results
Harms	Enhance reliability and quality of data	Information (instrument, recall and social desirability) bias: can reduce internal validity	Data collection
Discussion			
Limitations	N/A	N/A	–
Generalisability and applicability	Obtain buy-in from stakeholders	Can reduce implementation, sustainability and translation of results into practices	Background and setting
Interpretation	Identify the mechanisms underpinning the effect of the intervention	N/A	Analysis and results

*Abbreviations*: *CONSORT* Consolidated Standards of Reporting Trials, *N/A* not applicable

This provided the adapted framework for our thematic analysis of the in-depth interviews with scholars. Using this framework, we completed a thematic analysis in NVivo of the interview transcripts, aligning what scholars had said about the inherent usefulness of particular qualitative methods for the RCTs they had worked on with the RCT challenges in our framework. We then shared the draft framework with participants in two separate rounds in order to elicit feedback and facilitate group consensus on the alignment between RCT challenges and specific qualitative methods.

## Results

The aim of our analysis was to generate a list of innovative qualitative methods that help to address common methodological challenges of RCTs (Table 5). We present our results following the adapted CONSORT statement framework used to guide the analysis. Common methodological challenges are organised according to the merged categories, and potential qualitative methods that overcome each challenge are listed. Our results are presented according to seven categories: (1) background and setting, (2) intervention design and compliance, (3) participant recruitment and enrolment, (3) allocation of the intervention and randomisation, (5) participant follow-up, (6) data collection, and (7) analysis and results. Under each category, we have combined findings from the in-depth interviews and evidence from the literature review as a means of expanding the scope of the results and describing a more comprehensive methodological landscape.

**Table 5:** Framework for the integration of innovative qualitative methods into randomised controlled trials of complex health interventions

Categories adapted from CONSORT	Methodological Challenge	Threats to design or results	Type of bias	Validity affected	Qualitative research solution	Examples of qualitative methods
Background and setting	Ensure relevance of the problem to the context	Affects applicability, acceptability, sustainability and transferability of potential positive findings	N/A	External	To identify social, cultural, health, economic or political factors that might affect uptake and sustainability of positive results	Direct observation, ideally in-depth but if time or resources are limited, as part of a rapid ethnographic appraisal or Broad Brush Survey
	Obtain buy-in from stakeholders	Reduces translation and sustainability of results into changes in policy and practices	N/A	External	To overcome potential barriers to implementation and promote uptake of intervention	In-depth interviews with policymakers and stakeholders | Document analysis
Intervention design and compliance	Ensure cultural acceptability and practical feasibility of the intervention	Impacts adherence and increases number of drop-outs	Adherence Withdrawal	Internal	To tailor intervention in order to increase retention and adherence	Diary methods – via Interactive voice responses or SMS
Recruitment and enrolment	Guarantee representativeness of the sample and efficient recruitment methods	Risks achieving the required sample size to detect significant effect	Selection	Internal External	To determine the best possible recruitment method to reach target population	Community mapping | Spiral walks
Randomization and allocation	Guarantee balanced randomization	Reduces comparability between groups	Selection Confounding	Internal External	To identify contextual factors that can affect the effect of the intervention and reduce comparability between groups	Observation | Public randomization
Participant follow-up	Minimize the number of participants leaving the study	Enables unequal loss of participants between groups which can affect causal inference	Attrition Confounding (if differential attrition between trial arms)	Internal External	To prevent or understand reasons for loss to follow-up and improve retention strategies	Diary methods, mobile-based methods such as interactive voice response on SMS / WhatsApp | peer support for adherence
	Improve adherence to the intervention	Modifies the magnitude/direction of effect	Adherence	Internal	To understand reasons for non-adherence to the intervention	
Data collection	Enhance reliability and quality of data	Allows inconsistent or unreliable measurements which can affect the observed magnitude and direction of the effect	Instrument	Internal	To avoid or identify errors in the measurement and data collection process.	Co-designing measurement tools with participants | Qualitative tool validation
	Maintain objectivity during the data collection process	Threatens the validity of the data collected and/or measured to answer the objective	Instrument Recall Social desirability	Internal	To assess and validate the process of data collection.	FGD with prompts such as flashcards or images Pair interviews and role playing
Analysis and results	Identify the mechanisms underpinning the effect of the intervention	Limits an informed discussion of the results (negative or positive)	N/A	External	To triangulate the quantitative findings and identify contextual information that may have affected the results	Participatory Analysis methods

*Abbreviations*: *CONSORT* Consolidated Standards of Reporting Trials, *N/A* not applicable, *SMS* short message service.

### Background and setting

#### Challenge 1: Ensure relevance of the intervention to the context

Interviewees suggested that qualitative methods had an important role to play in ensuring that the design and adaptation of interventions correspond with local needs. They suggested a number of activities that could be completed either (a) before testing an intervention through a trial or (b) during the trial itself in order to ensure that there is fidelity and that the intervention remains relevant and acceptable. For instance, an assessment to identify the local health, social, cultural and political landscape was suggested as a means of providing important information about the need for an intervention within a particular context:

In global public health, most interventions are … delivered in quite complex settings and trying to understand the setting – how people go about their daily lives and then how that intervention is going to fit into those daily lives – is really important. I16

In the qualitative literature, direct observation is frequently mentioned as a particularly useful method for developing cultural understanding of context by enabling data to be gathered through first-hand or eyewitness experience [46]. Drawing on ethnographic techniques in anthropology, observation as a method often involves sustained immersion in the research setting for a long period [47], which is not always possible as part of formative research leading up to a trial or within the constraints of a process evaluation.

As an alternative, quicker approaches to ethnography were mentioned by interview participants, including social mapping, non-participant observation, and “Broad Brush Surveys”, which systematically gather data on communities in a period of 5–12 days [48]:

The point of [Broad Brush surveys] is to provide a narrow and rapid impression of the visible features of the communities and get a sense of the more invisible characteristics of the communities and how they might affect the particular research and interventions planned. I11

These rapid techniques draw on the advantages of ethnographic techniques while adhering to the limitations of a trial and the need for rapid results.

#### Challenge 2: Obtain buy-in from stakeholders, including practitioners and policymakers

If public health interventions undergoing trials are not informed by key stakeholders, including communities, community leaders, local policymakers and health providers at national and regional levels, they risk reduced sustainability and rejection by local actors. It is particularly important to spend time engaging communities to increase participation and recruitment and to promote ownership of the problem as well as the solution, as discussed during the interviews:

The ideal would be that we engage communities even before the trial is designed in order to decide what their priorities are and then use those priorities to develop the intervention. i18

The literature points to trials that have been stopped because of significant stakeholder ethical opposition [49]. Even interventions that are effective may not translate into improved practices or policies if they are not aligned with the current agenda of local governments or policymakers [50]. Therefore, it is important to consider and adapt to stakeholders’ concerns early on to increase subsequent dissemination and uptake of results.

Qualitative methods mentioned by interviewees to overcome these risks and promote take-up of the intervention include in-depth interviews with policymakers, observation and document analysis. Often, this was done not formally but informally as part of the early stages of a trial:

I talk to the stakeholders who are part of the PPI [Public Patient Involvement] groups, patients, and people who are delivering the intervention. I sit in on training to see what questions arise in training sessions and what people are obviously worried about when they’re thinking about delivering the intervention. I17

The literature similarly supports the use of in-depth semi-structured interviews as a means of understanding stakeholder perspectives because of the potential to produce data on people’s knowledge, understandings, interpretations, experiences and interactions [51]. With this information, one can enable the design of optimal, scalable interventions that could feasibly be translated into policy.

### Intervention design and compliance

#### Challenge 3: Ensure cultural acceptability and feasibility

Interventions that are not acceptable to the population or feasible in the local context will likely result in poor adherence and drop-out of participants, affecting both the magnitude of the effect and representativeness of the final sample in an RCT. Tailoring the intervention to increase its acceptability can also be challenging because of a reluctance of participants to share relevant information, social desirability bias [52] and power dynamics between researchers and participants [53]. An effective means of accessing crucial qualitative data on the acceptability and feasibility of an intervention mentioned by interviewees is diary methods:

They [fieldworkers] kept diaries because they were the people that were going on the wards every day and noticing the culture of the wards and able to see pertinent issues about the wards. I3

Recognised in the literature as a useful technique for obtaining observational data, diaries can be both qualitative and quantitative and are used to capture and record the details of time-sensitive and context-specific phenomena [54]. As they allow events to be recorded in their natural setting, diaries reduce the delay between an event and its recording and provide a level of privacy that should reduce potential bias [55].

Interviewees also mentioned the potential for mobile phone technology to be used to collect audio diaries through an interactive voice response process where participants record brief voice clips about their activities or perspectives when prompted:

So, [interactive voice response, or Short Message Service (SMS)] has been, I would say, key in helping us to track change over time but also gives us very practical information about what we need to do in terms of orienting the intervention to be most impactful. I15

Most of the research now is done on mobile phones […] it’s probably the best way to do research, to get large samples of anything. You use mobile phones here. I20

Such methods were also highlighted by interviewees as particularly useful with geographically hard-to-reach populations, tracking change in an intervention over time, or as a means of obtaining large sample sizes for data collection.

### Participant recruitment and enrolment

#### Challenge 4: Guarantee representativeness and improve recruitment

Developing recruitment techniques that reach the required sample size while reflecting the characteristics of the broader population is challenging:

We’ve recently been doing work in fishing communities, here, in Uganda, where it has been very much trying to get some sense of who spends time where in terms of, if you’re going to reach them with particular interventions, where are you going to find them? I10

Qualitative methods that capitalise on local knowledge can be invaluable for understanding complex social characteristics and differences that may lead to recruitment bias. To address this, both the literature and interviewees mentioned the usefulness of mapping methods, which bring together groups or communities to “map” assets, processes, or physical environments. These can be extremely useful in collecting information about resources, activities, and potential recruitment locations in a relatively short period [56]. A particularly effective technique mentioned by interviewees is mapping physical spaces and resources within a community by using transect or “spiral” walk methods to record how resources are distributed:

If you don’t walk through that area, you may not realise quite what’s going on. So the idea is to walk in a circle, facilitated, trying to come into contact with as many different people from as many different parts of a settlement as possible. You walk in a spiral. I10

Spiral walks use observation methods to identify people and places of significance to the research. The walk is usually in concentric circles from the centre to the outskirts of an area and can involve interviews *en route* [57]. The purpose of the circles is to cover all sections of a community; a transect walk in a straight line may leave out an area where a particular group of people live or work. Spiral walks were perceived by interviewees as providing a greater understanding of social structures in the community. However, in order to maximise the potential for recruitment, mapping methods should be combined with data from those implementing the recruitment procedures to assess the fit between the understanding of the social context and the way recruitment is currently being carried out within the trial.

### Randomisation and allocation of the intervention

#### Challenge 5: Guarantee comparability between groups

One of the main challenges of trials is to ensure balance across intervention arms in order to ensure that groups are comparable and that the difference between them can still be used to achieve causal interpretation [58]. Different underpinning cluster or individual characteristics can reduce the comparability between groups, affecting the effect size and directionality of the results in unmeasurable ways. Therefore, it is important to tease out contextual factors such as community characteristics or locations that can be stratified on during randomisation. As suggested by an interviewee:

We put these communities into different types and then allow that to be part of the randomisation process so rather than just randomising according to HIV prevalence or geographical location and so on. I11

Although considering contextual factors during randomisation can be difficult to achieve, it might also save a trial from losing validity. Participant observation at early stages of the study might allow researchers to witness a community’s dynamics and observe relevant information that can be used to identify strata for randomisation.

A participatory approach to identifying strata in collaboration with communities suggested during the interviews was to do public randomisation:

You know, usually it’s just the scientists going into a room and using a random number generator. But putting the clusters together and also making sure that the clusters were balanced really required the community participation because they understand the agencies involved. Then even on the randomisation inside, they helped ... we had a really great statistician who did a group facilitated exercise to come up with the randomisation. I4

Involving communities in the process of identifying strata to be used in randomisation in this way can help to ensure that the randomisation process considers potential similarities and differences in groups that are not directly observable by the research team. This can be a particularly useful method when there are a relatively small number of clusters to be randomly assigned.

### Participant follow-up

#### Challenge 6: Minimise the number of subjects leaving the study. Improve adherence to treatment or intervention

Complex health interventions often address highly challenging health problems among marginalised populations through multiple intervention components. The challenging lives of targeted populations combined with the complexity of the intervention itself often magnify the number of people leaving the study and reduce potential adherence for those who stay. To effectively gather data on loss to follow-up and adherence, an interviewee recommended monitoring participants and adapting the intervention over time:

…we always explicitly said that we’re going to continually measure how our intervention is working or not working in terms of whether people like it, whether people are using it, so that we can adapt our intervention as we go. I8

Interviewees also mentioned the need to maintain constant communication with participants and the advantages of doing so through highly available methods, such as SMS or WhatsApp technologies:

Wherever the population is widely using smart phones I would say, it’s worth [collecting data] on smart phones… it’s just easy to access people that way, and you can access people who you wouldn’t otherwise be able to access… you know, very specific groups. I20

The advantages of mobile communication technologies are also emphasised in the literature for their ability to provide a closer follow-up with participants as well as acting as an alternative data collection source [59].

### Data collection

#### Challenge 7: Enhance reliability and quality of the data

Data reliability in RCTs can be affected through several mechanisms, some related to the process and others to the intrinsic nature of the data gathered. On one hand, collecting data under real-life conditions can affect the pre-standardized process of data collection. On the other hand, trials that evaluate complex public health interventions may also rely on outcomes that are subject to social desirability bias, which compromises objectivity in the measurement of outcomes.

One means of addressing this and increasing the reliability and validity of qualitative data mentioned by interviewees is the use of community-based participatory research (CBPR) methods. CBPR collaboratively involves community stakeholders, such as community groups and organisations and members of the community, at all stages of the research process [60]. The advantages of the deep involvement of participants is the potential for participants to forget that they are being researched:

If they [people] are involved in an activity with a task that’s group-based, I think that they forget that they’re involved in research, and so you might get slightly more accurate insights into people’s perceptions and behaviours because they don’t feel quite so much like they’re on show. I21

The literature also emphasises the potential of CBPR methods and in particular measurement tools that are co-designed with participants to ensure content validity, construct validity and consensual validity [61]. However, iterative tool development is understood as a long-term process, which should take place over multiple sessions and consultations with stakeholder groups within the community [61].

#### Challenge 8: Maintain objectivity during the data collection process

Many complex interventions are aimed at changing behaviours at an individual level and collect data based on self-reports, which can hinder the collection of valid data when there are complex social structures influencing people’s behaviours. An example of this challenge was introduced in one of the interviews:

So, our quantitative data showed very high rates of handwashing, but our observations showed that the reality was quite different. People often didn’t have soap in the house or only washed their hands with water, but they actually said something else in the quantitative interview. So, it gave us a better contextual understanding of the lives of those families and the structural barriers that they faced to changing their behaviour, and also, the limitations of our quantitative data, which was useful. I12

Although many RCTs select “hard” endpoint variables to ensure the identification of effect, measuring the process remains predominantly “soft”. It is a challenge for RCTs to find strategies to collect high-quality, valid process data. The susceptibility to potential biases such as social desirability or recall bias increases in complex public health interventions, and identifying the best qualitative methodology to capture issues with validity becomes imperative to maintaining the objectivity of the study.

To prevent socially desirable responses from participants, interviewees mentioned the potential for embedding well-structured activities into focus group discussions to help access more reliable data. For instance, flashcards or images-as-prompts can be used to access the immediate reactions and understandings of participants, to identify social taboos, and to understand misconceptions about the intervention that could create challenges for the RCT. This same idea is evident in literature that mentions the advantages of having participants organise or “pile sort” images to assess the importance of certain ideas or issues for a group or to gain an understanding of local priorities [62]. Asking participants to order events as they believe they would or should happen can gauge a sense of people’s daily lives or processes of care-seeking. Interviewees also mentioned the potential of interviewing young people in pairs or with friends or using role-play or song to address shyness or to obtain valuable information from younger participants:

We’ve tried friendship pair interviews where we’ve tried to get two friends to talk things through with the idea that if they’re with somebody who they feel very comfortable with the social desirability bias might be less and the friend might have some insights into the person’s behaviour. I2

All of these activities offer an opportunity to more effectively access the psychological processes underlying perceptions and opinions of participants.

### Analysis and results

#### Challenge 9: Identify the mechanisms underpinning the effect of the intervention

Although trials are useful for evaluating the effect of a designed intervention, they fall short in explaining the mechanisms behind an intervention’s effect. Qualitative data can help address this, as acknowledged in the MRC guidance on process evaluation [42] and discussed in an interview:

…the quantitative piece will really help us understand the scale of the impact but the qualitative we’re feeding in to understanding kind of the practices and if it worked, how it worked and why it worked and we’re definitely very interested once we have the endline [data], both quantitative and qualitative to do a lot of cross-triangulation so that those two datasets kind of speak to each other. I1

Without qualitative data, trials that show little effect of an intervention will not have an explanation of why this may have occurred or which component failed. This is especially important in trials evaluating behaviour change, in which interpretations of what has changed may differ between participants and researchers. One interviewee mentioned the use of “visual participatory analysis” to understand community perceptions of the results of the trial. This method involves engaging participants in a community-based focus group discussion about the meaning of the quantitative results. During the focus groups, the participants discuss a series of graphic representations of the study’s results. These qualitative data then provided additional data that were used to inform the interpretation of the quantitative results. Where an intervention is complex and the impacts on people’s lives are likely to be wide-ranging, this method offers a potential means of eliciting different perspectives on the intervention and its effects. It moves beyond recommendations that results be disseminated to local communities [63] by offering a means of ensuring that the qualitative findings from this process are integrated with the analysis of the quantitative findings from the trial.

## Discussion

Our findings present a new framework for integrating qualitative methods into RCTs of complex interventions with the aim of contributing to methodological discussions about the value of diverse qualitative methods for understanding complex interventions in global health and addressing the challenges of RCTs. Similar to others, our framework is a process rather than a temporal framework but has the added value of situating the benefits of using qualitative methods against the specific challenges of RCTs. This contributes directly to calls for innovation in the use of RCT methodologies to evaluate complex health interventions and the challenges they face in maintaining internal and external validity [64].

The findings of this study point specifically to the usefulness of observational and participatory methods for trials of complex public health interventions, offering a novel contribution to the broader literature about the need for mixed methods approaches. The findings highlight the benefits of using methods such as diaries, mapping techniques, and observational spiral walks to understand the social, political, and cultural context surrounding both interventions and trials. The findings also outline the benefits of using community participation to inform key trial design decisions drawing on methods such as public randomisation and “community-based participatory research” as part and parcel of trials. Finally, the findings highlight the advantages of using observation and participatory approaches, such as “visual participatory analysis”, to understand the effects of an intervention. This is a radical departure from the use of endline interviews with participants or focus group discussions as part of a mixed methods process evaluation. Scholars have recognised the constraints of collecting brief qualitative data during a trial and the limited understanding of the process of the intervention this generates [65]. The methods suggested as part of this study provide a means of harnessing the rich potential of qualitative research to understand context, to subsequently ensure that the intervention is the best for this context, and finally to understand its effects.

In a study of how qualitative and quantitative data are combined in RCTs, the QUAlitative Research in Trials (QUART) study, researchers found that those who take an “integrated methods approach” also see qualitative research as essential to the trial and as producing evidence related to the “real world” [3]. The QUART study therefore recommends that researchers design and implement “studies not trials”, with the outcomes of the qualitative research being “central to the team’s thinking” (page 124). In practice, the implementation of “studies” rather than “trials” requires researchers to adopt a neutral approach to methods. This essentially means selecting the best method for the research question posed rather than making presumptions about which methods are best based on a hierarchy of evidence [66]. For example, if the question is about whether people have changed health-related beliefs, then the research team potentially has multiple methods to choose from, which may include qualitative interviews, focus group discussions, photovoice, quantitative surveys, or a combination of these methods. The selection of which method is best should be driven by identifying the method that is best for answering the specific question for a particular context [67]. This approach to method neutrality may be particularly useful for complex public health interventions with strong theoretical models. Trials that are driven by theories of change or logic models have a series of research questions that may be posed alongside each stage of the model in an enumerated fashion. In a method-neutral approach, each of these stages requires that the research team identify the most appropriate methods on the basis of the question and the context. The diverse toolbox of qualitative methods suggested as part of our framework helps to foster such an approach.

The findings presented in this article have also shown how a diverse toolkit of qualitative methods can enable appropriate adjustments during intervention development to create more sustainable interventions [7]. It can determine the relevance of a trial to participants, hence ensuring successful recruitment and follow-up, and can also identify behaviours and biases of those recruiting participants to a trial and perceptions of participants that may explain differences in outcome [30]. Finally, qualitative research can indicate why successful interventions might not work in the real world and find the best ways to ensure that findings are translated to policy by establishing how results may be received by relevant parties, enhancing the utility, effectiveness and applicability of findings from RCTs [30, 68]. Regardless of whether we continue to use standardised randomised trials or seek out more pragmatic designs, we need to move beyond the division between quantitative and qualitative methods and see public health evaluation itself as a form of mixed methods research. To accomplish this, the purpose and value of qualitative methods need to be highlighted within both trial protocols and published studies, and the integration of qualitative research needs to be considered at the earliest stages of grant proposal development [14].

Implicit in the integration of quantitative and qualitative research in the evaluation of complex public health interventions is a need for greater engagement between disciplines with strong qualitative or quantitative traditions, such as anthropology and epidemiology. This is already happening to a certain extent with interdisciplinary relationships between anthropologists and epidemiologists contributing to more nuanced understandings of human behaviour and interventions better suited for local contexts [69]. However, experiences of working collaboratively across disciplines is not without its challenges and adopting a method-neutral approach as discussed above requires an acceptance of methodology as a socially constructed tool used to “interpret” rather than “observe” reality [70]. This will require changes by all research actors, including scholars, universities, funders and those publishing research results.

Along similar lines, a fundamental change in the publishing bias of medical journals which favours brief quantitative articles is needed to bring method-neutral and transdisciplinary approaches to public health evaluation research [71]. In 2016, the current bias towards publishing quantitative articles led to an open letter to the *British Medical Journal* calling for it to “move beyond a ‘quantitative strong, qualitative weak’ stance” and to stop “rejecting qualitative research on the grounds of low priority” [72] (page 2). Both the QUART study (in interviews) and our own qualitative interviews found a culture in which qualitative research is not cited as frequently as quantitative research and is often not published in high-impact journals [72].

### Limitations of this study

Given the foundation of this study in expert opinions and experiences, our findings are naturally influenced by the types of complex health interventions the experts interviewed were involved in. They may not be equally applicable to the wide variety of complex interventions currently being tested using different RCT designs, including those testing systems versus drugs or devices, treatment-focused versus prevention-focused interventions, and cluster randomised versus individually randomised designs. The methods we have discussed as part of our framework are not an exhaustive list, and we would encourage researchers to investigate alternative methods from different disciplinary perspectives that meet their specific trial needs. Our presentation of different qualitative methods does not consider the ethical implications of using these methods and this would need to be assessed as part of the specific aims and objectives of the trial being undertaken.

## Conclusions

We argue that it is through dialogue and recognition of the complementarity of disciplines that the expansion of successful health-related interventions will be achieved. We have contributed to this dialogue by presenting innovative ways in which qualitative research can be integrated into RCTs to improve research quality and to increase health impact. We hope this encourages researchers to enter into new studies with a broader understanding of what counts as “evidence” for impact evaluations and how qualitative research can strengthen the validity and usefulness of RCT research for the future.

## Data Availability

The data used and analysed during the current study are available from the corresponding author on reasonable request.

## Abbreviations

CBPR: Community-based participatory research
CONSORT: CONsolidated Standards Of Reporting Trials
MRC: Medical Research Council
QUART: QUAlitative Research in Trials study
RCT: Randomised controlled trial
SMS: Short message service

## References

[1] Rapport F, Storey M, Porter A, Snooks H, Jones K, Peconi J (2013). Qualitative research within trials: developing a standard operating procedure for a clinical trials unit. Trials.

[2] O’Cathain A, Thomas KJ, Drabble SJ, Rudolph A, Hewison J (2013). What can qualitative research do for randomised controlled trials? A systematic mapping review. BMJ Open.

[3] O'Cathain A, Thomas KJ, Drabble SJ, Rudolph A, Goode J, Hewison J. Maximising the value of combining qualitative research and randomised controlled trials in health research: the QUAlitative Research in Trials (QUART) study--a mixed methods study. Health Technol Assess. 2014;18(38). 10.3310/hta18380 PMC4781055 24914457

[4] Murtagh MJ, Thomson RG, May CR, Rapley T, Heaven BR, Graham RH (2007). Qualitative methods in a randomised controlled trial: the role of an integrated qualitative process evaluation in providing evidence to discontinue the intervention in one arm of a trial of a decision support tool. Qual Saf Health Care.

[5] Lewin S, Glenton C, Oxman AD (2009). Use of qualitative methods alongside randomised controlled trials of complex healthcare interventions: methodological study. BMJ.

[6] Pope C, Mays N (1995). Qualitative research: reaching the parts other methods cannot reach: an introduction to qualitative methods in health and health services research. BMJ.

[7] Jansen YJ, Foets MM, de Bont AA (2009). The contribution of qualitative research to the development of tailor-made community-based interventions in primary care: a review. Eur J Pub Health.

[8] Moore G, Audrey S, Barker M, Bond L, Bonell C, Cooper C (2014). Process evaluation in complex public health intervention studies: the need for guidance. J Epidemiol Community Health.

[9] Campbell M, Fitzpatrick R, Haines A, Kinmonth AL, Sandercock P, Spiegelhalter D, et al (2000). Framework for design and evaluation of complex interventions to improve health. BMJ.

[10] Craig P, Dieppe P, Macintyre S, Michie S, Nazareth I, Petticrew M (2013). Developing and evaluating complex interventions: the new Medical Research Council guidance. Int J Nurs Stud..

[11] Power R, Langhaug L, Nyamurera T, Wilson D, Bassett M, Cowan F (2004). Developing complex interventions for rigorous evaluation—a case study from rural Zimbabwe. Health Educ Res..

[12] Petticrew M. (2011). When are complex interventions 'complex'? When are simple interventions 'simple'?. The European Journal of Public Health.

[13] Greenhalgh T, Papoutsi C. Studying complexity in health services research: desperately seeking an overdue paradigm shift. BMC Med. 2018;16:95. 10.1186/s12916-018-1089-4. 10.1186/s12916-018-1089-4 PMC6009054 29921272

[14] Drabble SJ, O’Cathain A, Thomas KJ, Rudolph A, Hewison J (2014). Describing qualitative research undertaken with randomised controlled trials in grant proposals: a documentary analysis. BMC Med Res Methodol..

[15] Ravallion M. Should the randomistas rule?. The Economists' Voice. 2009;6(2).

[16] Mowat R, Subramanian SV, Kawachi I (2018). Randomized controlled trials and evidence-based policy: A multidisciplinary dialogue. So Sc Med (1982).

[17] Elford J, Bolding G, Sherr L (2001). Peer education has no significant impact on HIV risk behaviours among gay men in London. AIDS.

[18] Kinsman J, Nakiyingi J, Kamali A, Carpenter L, Quigley M, Pool R, Whitworth J (2001). Evaluation of a comprehensive school-based AIDS education programme in rural Masaka, Uganda. Health Educ Res..

[19] Wight D, Raab GM, Henderson M, Abraham C, Buston K, Hart G, Scott S (2002). Limits of teacher delivered sex education: interim behavioural outcomes from randomised trial. BMJ.

[20] Craig P, Dieppe P, Macintyre S, Michie S, Nazareth I, Petticrew M: Developing and evaluating complex interventions: updating Medical Research Council guidance to take account of new methodological and theoretical approaches. Edited by MRC. UK: Medical Research Council; 2019.

[21] Montgomery C (2016). From standardization to adaptation: Clinical trials and the moral economy of anticipation. Sci Cult..

[22] Pallmann P, Bedding AW, Choodari-Oskooei B, Dimairo M, Flight L, Hampson LV, Holmes J, Mander AP, Odondi L, Sydes MR (2018). Adaptive designs in clinical trials: why use them, and how to run and report them. BMC Med..

[23] Schwartz D, Lellouch J (2009). Explanatory and pragmatic attitudes in therapeutical trials. J Clin Epidemiol..

[24] Hawe P, Shiell A, Riley T (2004). Complex interventions: how “out of control” can a randomised controlled trial be?. BMJ.

[25] Hotopf M (2002). The pragmatic randomised controlled trial. Adv Psychiatr Treat.

[26] Chalmers I, Glasziou P (2009). Avoidable waste in the production and reporting of research evidence. Lancet.

[27] Glasziou P, Altman DG, Bossuyt P, Boutron I, Clarke M, Julious S (2014). Reducing waste from incomplete or unusable reports of biomedical research. Lancet.

[28] Yordanov Y, Dechartres A, Porcher R, Boutron I, Altman DG, Ravaud P (2015). Avoidable waste of research related to inadequate methods in clinical trials. BMJ.

[29] Sandelowski M (1996). Using qualitative methods in intervention studies. Res Nurs Health.

[30] Flemming K, Adamson J, Atkin K (2008). Improving the effectiveness of interventions in palliative care: the potential role of qualitative research in enhancing evidence from randomized controlled trials. Palliat Med..

[31] Bradley F, Wiles R, Kinmonth A-L, Mant D, Gantley M (1999). Development and evaluation of complex interventions in health services research: case study of the Southampton heart integrated care project (SHIP). BMJ.

[32] Mannell Jenevieve, Davis Katy (2019). Evaluating Complex Health Interventions With Randomized Controlled Trials: How Do We Improve the Use of Qualitative Methods?. Qualitative Health Research.

[33] Bogner A (2012). The paradox of participation experiments. Sci Technol Hum Values.

[34] Wiles R, Crow G, Pain H (2011). Innovation in qualitative research methods: A narrative review. Qual Res..

[35] Okoli C, Pawlowski SD (2004). The Delphi method as a research tool: an example, design considerations and applications. Inf Manag..

[36] Rowe G, Wright G (2011). The Delphi technique: Past, present, and future prospects—Introduction to the special issue. Technol Forecast Soc Chang..

[37] Gale NK, Heath G, Cameron E, Rashid S, Redwood S (2013). Using the framework method for the analysis of qualitative data in multi-disciplinary health research. BMC Med Res Methodol..

[38] Schultz KF (2010). CONSORT statement: updated guidelines for reporting parallel group randomised trials. BMC Med..

[39] Moher D, Hopewell S, Schulz KF, Montori V, Gøtzsche PC, Devereaux P (2012). CONSORT 2010 explanation and elaboration: updated guidelines for reporting parallel group randomised trials. Int J Surg..

[40] Puffer S, Torgerson D, Watson J (2003). Evidence for risk of bias in cluster randomised trials: review of recent trials published in three general medical journals. BMJ.

[41] Datta J, Petticrew M (2013). Challenges to evaluating complex interventions: a content analysis of published papers. BMC Public Health.

[42] Moore GF, Audrey S, Barker M, Bond L, Bonell C, Hardeman W (2015). Process evaluation of complex interventions: Medical Research Council guidance. BMJ.

[43] Campbell MJ (2014). Challenges of cluster randomized trials. J Comp Eff Res.

[44] Ivers NM, Taljaard M, Dixon S, Bennett C, McRae A, Taleban J (2011). Impact of CONSORT extension for cluster randomised trials on quality of reporting and study methodology: review of random sample of 300 trials, 2000-8. BMJ.

[45] Giraudeau B, Ravaud P (2009). Preventing bias in cluster randomised trials. PLoS Med..

[46] Harvey SA (2018). Observe Before You Leap: Why Observation Provides Critical Insights for Formative Research and Intervention Design That You’ll Never Get From Focus Groups, Interviews, or KAP Surveys. Glob Health Sci Pract..

[47] Mays N, Pope C (1995). Qualitative research: observational methods in health care settings. BMJ.

[48] Bond V, Ngwenya F, Murray E, Ngwenya N, Viljoen L, Gumede D (2019). Value and limitations of Broad Brush Surveys used in Community-Randomized Trials in Southern Africa. Qual Health Res..

[49] Newman PA, Rubincam C, Slack C, Essack Z, Chakrapani V, Chuang D-M (2015). Towards a science of community stakeholder engagement in biomedical HIV prevention trials: an embedded four-country case study. PLoS One.

[50] Heneghan C, Goldacre B, Mahtani KR (2017). Why clinical trial outcomes fail to translate into benefits for patients. Trials.

[51] Lewis-Beck MBA, Liao TF. The Sage encyclopedia of social science research methods. USA: SAGE Publications, Inc.; 2004.

[52] Latkin CA, Mai NVT, Ha TV, Sripaipan T, Zelaya C, Le Minh N (2016). Social desirability response bias and other factors that may influence self-reports of substance use and HIV risk behaviors: a qualitative study of drug users in Vietnam. AIDS Educ Prev..

[53] Karnieli-Miller O, Strier R, Pessach L (2009). Power relations in qualitative research. Qual Health Res.

[54] Hyers Lauri (2018). Diary Methods.

[55] Lavrakas PJ. Encyclopedia of survey research methods (Vol. 2). Thousand Oaks: SAGE; 2008.

[56] Tock. Shortwork [Internet]. Liverpool: Hull and East Riding Participatory Appraisal Network. 2001. Available from: http://shortwork.org.uk/participatory-research/an-introduction-to-participatory-appraisal/.

[57] Ngwenya N, Gumede D, Shahmanesh M, McGrath N, Grant A, Seeley J (2018). Community perceptions of the socio-economic structural context influencing HIV and TB risk, prevention and treatment in a high prevalence area in the era of antiretroviral therapy. Afr J AIDS Res.

[58] Esserman D, Allore HG, Travison TG (2016). The Method of Randomization for Cluster-Randomized Trials: Challenges of Including Patients with Multiple Chronic Conditions. Int J Stat Med Res..

[59] Singh G, Manjunatha N, Rao S, Shashidhara HN, Moirangthem S, Madegowda RK (2017). Use of mobile phone technology to improve follow-up at a community mental health clinic: A randomized control trial. Indian J Psychol Med..

[60] Israel BA, Schulz AJ, Parker EA, Becker AB (1998). Review of community-based research: assessing partnership approaches to improve public health. Annu Rev Public Health.

[61] Flicker S, Guta A, Larkin J, Flynn S, Fridkin A, Travers R (2010). Survey design from the ground up: Collaboratively creating the Toronto Teen Survey. Health Promot Pract..

[62] Colucci E (2007). “Focus groups can be fun”: The use of activity-oriented questions in focus group discussions. Qual Health Res..

[63] Fernandez CV, Kodish E, Weijer C (2003). Informing study participants of research results: an ethical imperative. IRB.

[64] Gillies C, Freemantle N, Grieve R, Sekhon J, Forder J (2016). Advancing quantitative methods for the evaluation of complex interventions.

[65] Leeming D, Marshall J, Locke A (2017). Understanding process and context in breastfeeding support interventions: The potential of qualitative research. Matern Child Nutr..

[66] Borgerson K (2009). Valuing evidence: bias and the evidence hierarchy of evidence-based medicine. Perspect Biol Med..

[67] Creswell JW (2015). A Concise Introduction to Mixed Methods Research.

[68] Donovan J, Mills N, Smith M, Brindle L, Jacoby A, Peters T (2002). for the ProtecT Study Group: Improving design and conduct of randomised controlled trials by embedding them in qualitative research: ProtecT (prostate testing for cancer and treatment) study. BMJ.

[69] Trostle JA, Sommerfeld J (1996). Medical anthropology and epidemiology. Annu Rev Anthropol..

[70] Béhague DP, Gonçalves H, Victora CG (2008). Anthropology and epidemiology: learning epistemological lessons through a collaborative venture. Cien Saude Colet.

[71] Camlin CS, Seeley J (2018). Qualitative research on community experiences in large HIV research trials: what have we learned?. J Int AIDS Soc.

[72] Greenhalgh T, Annandale E, Ashcroft R, Barlow J, Black N, Bleakley A (2016). An open letter to The BMJ editors on qualitative research. BMJ.

